# Wrongness and Blame Judgments and Their Dynamics: Toward a Three-Input Processing Model of Moral Judgment

**DOI:** 10.5334/irsp.868

**Published:** 2024-11-11

**Authors:** Aurore Gaboriaud, Flora Gautheron, Jean-Charles Quinton, Annique Smeding

**Affiliations:** 1 Univ. Grenoble Alpes, Univ. Savoie Mont Blanc, LIP/PC2S, F-38000 Grenoble, France; 2 Univ. Grenoble Alpes, CNRS, Grenoble INP, LJK, F-38000 Grenoble, France; 3 Univ. Savoie Mont Blanc, Univ. Grenoble Alpes, LIP/PC2S, F-73000 Chambéry, France

**Keywords:** decision process, moral judgment, intent, outcome, causality, mouse-tracking

## Abstract

In moral psychology, several approaches to moral judgments coexist, with sometimes contradictory results for different types of judgments. In the current research, we combine two views of moral judgment into a novel three-input processing model. As a first empirical test of this model, the present research investigates the influence of these three classic inputs (i.e., intent, outcome, and causality) on wrongness and blame judgments as well as their underlying dynamics. This preregistered experiment (*N* = 145) re-uses an adapted mouse-tracking paradigm to analyze these influences over time. Results on final judgments replicate the effects of intent, outcome, and causality, as well as partial evidence for their interaction effects. Mouse trajectory analysis further refines these interaction effects, including evidence for differential dynamics for blame versus wrongness judgments. However, this study does not reveal clear differential weight for intent and outcome inputs in blame versus wrongness judgments. Discussion focuses on the evidence supporting but also contradicting the proposed three-input processing model and insists on the importance of distinguishing between final judgments and underlying dynamics.

In daily life situations, we often consider wrongdoings (like murdering, lying, or cheating) to be punishable or condemnable in proportion to how wrong they are: The worse the act, the harsher the judgment. However, think a moment about the following scenario: You find out that the neighbor you’re very close to has cheated on his tax return for a rather impressive amount. You would probably judge this act morally wrong. But would you blame your neighbor as harshly if you knew it was to pay for his daughter’s higher education, otherwise unaffordable? This example illustrates that wrongness judgments do not necessarily overlap with blame judgments and points at possible different underlying mechanisms.

The current study combines two views of moral judgment into a novel processing model and provides an initial empirical test of it as a function of judgment types. We therefore investigated in moral scenarios the influence of three traditional inputs (i.e., intent, outcome, and causality) on wrongness and blame judgments and their underlying dynamics.

## Intent and Causal Information in Moral Judgments

Plural factors have been demonstrated to play a role in how we judge agents’ immoral actions, such as those of our hypothetical neighbors. In particular, agents’ intent—defined by Cushman (2008) as the combination of agents’ beliefs and desires—weighs heavily in moral decisions or judgments (e.g., Cushman 2008; Cushman et al. 2013; Gaboriaud et al. 2022; Nobes et al. 2017; Young & Tsoi 2013). According to the literature, and consistent with an intuitive reasoning approach, intentional immoral acts are perceived as much worse morally speaking than accidentally perpetrated ones (e.g., Cushman 2008; Leloup et al. 2018; Young et al. 2007). Besides intent, the action outcome for the victims—in line with the consequentialist moral tradition (e.g., Gawronski & Beer 2017) and with the moral luck literature (e.g., Kneer & Machery 2019)—is another key factor in moral judgments (e.g., Cushman 2008; Kneer & Skoczeń 2023). Immoral acts followed by a harmful outcome are perceived as morally worse than acts without any consequence or a neutral outcome.

Yet another key factor is the agent’s causal involvement in the outcome (i.e., whether the outcome is caused by the agent himself or herself or by another independent source) (e.g., Cushman 2008; Gaboriaud et al. 2022). Cushman (2008) demonstrated that even if intent often explains a preponderant part of moral judgments variance, outcome and causality—what he summarized as ‘causal information’—also account for significant variability, especially for blame and punishment. More recent research further provides evidence in this direction: People judge immoral acts more harshly when directly caused by the agent rather than by another source (Gaboriaud et al. 2022).

Some evidence indicates that intentional and causal factors may interact. Cushman (2008) identified an interaction effect between intentional and causal components when asking participants about specific types of judgments (i.e., blame and punishment in Experiment 3). Gaboriaud et al. (2022) replicated the intent-by-causality interaction effect on judgments of punishment. People gave indeed more weight to the intent feature when the agent’s behavior was the causal origin of the harmful consequences. The effect of intent does not seem to be influenced by the outcome (Cushman 2008; Gaboriaud et al. 2022; Leloup et al. 2018), except in some research with judgments of permissibility (Young et al. 2007). Whether intent, causality, and possibly outcome interact, and which weight they trigger during the decision process may therefore depend on the type of judgment at stake (i.e., punishment, blame, wrongness, or permissibility). But this has not been systematically tested in the literature so far with a dynamic methodology, a within-pp design, and many more scenarios than usually used.

Cushman indeed demonstrates in his article (2008) the existence of two distinct and competitive processes in moral judgments, one based on the inference of the agent**’**s mental states (belief and desire), and the other based on the inference of causal information (about the outcome of the situation and the agent**’**s causality in this outcome). According to this model, wrongness judgments are not supposed to be influenced by causal information whereas punishment and blame do. More so, the intent weight on wrongness judgments is not supposed to vary depending on the occurrence of a harmful outcome, whereas it is supposed to be the case in punishment or blame judgments (at least when interacting with the causal part). Cushman’s studies (2008) are, however, limited by a between-participant design and a limited number of scenarios. Gaboriaud et al. (2022) did use a paradigm allowing to resolve these limitations but only explored one type of moral judgment (i.e., punishment).

## Integrating Moral Judgment Types into a Novel and Dynamic Three-Input Processing Model

Moral psychology literature is abundant but also puzzling as many approaches coexist (as suggested in the preface of the *Atlas of Moral Psychology*, edited by Gray and Graham in 2018), with sometimes contradictory results for different types of moral judgments. Several authors proposed theoretical models to better grasp and classify the world of morality. Regarding the question of the different types of judgment and decision processes in morality, it has recently been summarized by Malle (2021), who offers a typology according to the ‘typical objects of judgment, the information they process, and their social functions’ (2021, p. 294). Malle (2021) distinguishes four types of moral judgments, classified according to their cognitive processing complexity: evaluations, norm judgments, wrongness judgments, and blame judgments. Cushman’s two-process model (2008) focuses for its part on information processing within four types of moral judgments: wrongness, permissibility, punishment, and blame judgments. These classifications overlap at some point but have not yet been integrated. This is one of our aims via the model we propose in the present research, along with an initial empirical test of it investigating the influence of three key factors (as mentioned above) on various types of moral judgment.

In Cushman’s model (2008), blame and punishment judgments should trigger similar cognitive processes involving the analysis of both intentional and causal features into two distinct and competitive processes. Whereas wrongness and permissibility judgments should trigger processes almost entirely relying on the analysis of intent and not (or to a lesser extent) on causal information (Cushman 2008). Some evidence supports Cushman’s two-process model of moral judgment, especially for judgments of punishment (Gaboriaud et al. 2022; Leloup et al. 2018). For instance, Gaboriaud et al. (2022) found that when punishing the agent for a wrong action, people rely both on intent and causal information (encompassing the outcome and the causal involvement of the agent), with intent and causality interacting. Furthermore, using computer mouse-tracking to trace temporality, the authors found that intent influenced the decision of punishment very early in the process because it was seen first. More interestingly were the dynamics of outcome and causality: Outcome influenced the decision process from around the middle of the trials onwards, whereas causality information had a significant influence earlier in the decision process (around 20% of the trials). As we chose to rely on the same factors for the current study investigation (i.e., intent, outcome, causality), we expected to replicate these temporal tendencies, although that was not the main aim of the present research.

Departing from Cushman’s (2008) grouping of blame and punishment judgments, recent findings suggest that blame judgments are rather different from punishment which would better reflect a moral action than a moral judgment per se—hence going beyond the mere judgment and engaging a behavioral aspect (Malle 2021). Blame would therefore be more assimilated to wrongness or permissibility judgments for some authors, such as Kneer and Machery (2019). But others clearly differentiate blame from wrongness judgments, notably because they do not target the same object (i.e., the agent vs. the act respectively, see Malle et al. 2014). Kneer and Machery (2019) found a robust effect of outcome (i.e., moral luck effect) for punishment judgments but not for the other judgment types in a within-participant design. However, they found this effect for all types of judgment with a between-participant design, but it was almost entirely due to negligence ascriptions (i.e., the unlucky agent is seen as more negligent than the lucky one), not causal information per se.

**Figure 1:**
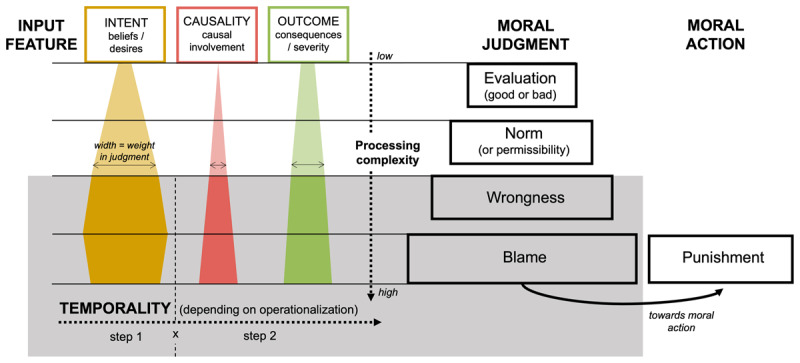
Proposed Three-Input Processing Model of Moral Judgment. *Note*. This three-input processing model is meant as an integration of Cushman’s (2008) and Malle’s (2021) models of moral judgment and is represented here with its operationalization in the current study. The right side of the figure contains the various judgment types classified depending on their respective processing complexity. On the left side of the figure, the weight of input features is displayed depending on the type of moral decision process involved, which may change when navigating through the hierarchy of judgments and actions (varying width). Light colors represent input weights that have not been tested in the current research whereas dark-colored areas stand for weights of input features that are currently tested in the present research (on wrongness and blame judgments only). The grey area with the temporal line corresponds to the current study operationalization with a specified order of presentation for the various features (i.e., intent is presented first, then outcome and causality in a second step).

In front of these puzzling tendencies and to integrate these different approaches and findings regarding types of moral judgments and underlying information processing, we introduce and illustrate in Figure 1 a three-input processing model of moral judgment, inspired by both Malle’s (2021) and Cushman’s (2008) approaches. On one hand, Cushman’s model (2008) does not integrate all types of judgment described by Malle (2021) and does not qualify the weight of each feature (i.e., intent or causal information) depending on judgment types and their underlying processing complexity (i.e., how much information is considered). On the other hand, Malle’s (2021) classification does not take into account the type of information they trigger. Furthermore, none of these frameworks considers judgment dynamics, that is, the temporal integration of intent and causal information.

The present three-input processing model of moral judgment therefore combines Malle’s classification along with intent and causal information as inputs for each judgment type as in Cushman (2008). It further differentiates causal information between outcome and causality features, along with input weights as a function of judgment type and their dynamics. We use the *three-input processing* designation for this model to reflect the three inputs considered (i.e., intent, causality, and outcome). Another key precision is that we do neither pretend via the current model to identify the various neural processes at play, nor to demonstrate the existence of three competitive processes (one for each input) in Cushman’s meaning of a process (2008), but more to tackle the intertwining of several information inputs in play during moral judgments.

To provide a first empirical test of the proposed three-inputs processing model (Figure 1), the present research investigates the influence of the agent’s intent (taking only the belief component from Cushman’s conceptualization in 2008, as demonstrated to be of main importance compared to the desire component), the outcome (i.e., the mere presence of a harmful consequence), and the causality (i.e., whether the agent is causally responsible for the consequence to occur) inputs on wrongness and blame final judgments and underlying dynamics. Indeed, findings consistent with this model already exist for punishment judgments and dynamics (Gaboriaud et al. 2022), hence the new focus on blame. In addition, wrongness judgments are more frequently investigated compared to permissibility judgments, in particular by Cushman (2008) when studying the intervention of causality on this type of moral judgment (study 3), and by Leloup et al. (2018, study 2) when comparing the effects of type of judgment order. Blame and wrongness judgments were therefore the best candidates for the current study. Given the controversies in the literature about their differentiation (Cushman 2008; Kneer & Machery 2019; Malle et al. 2014), it allows here to examine which and when features are processed during both types of judgments.

## Research Overview

The present research aims to provide a first empirical test of the proposed three-input processing model of moral judgment illustrated in Figure 1, integrating both Cushman’s (2008) and Malle’s (2021) frameworks. First, we will test the influence of intent, outcome, causality, and their interaction effects on wrongness and blame judgments. Leloup et al. (2018) partly tested these effects for punishment and wrongness judgments but did not manipulate causality. Gaboriaud et al. (2022) did manipulate causal information—distinguishing cause and outcome (as in Cushman, 2008, study 3)—but only focused on judgments of punishment. In contrast to Leloup et al. (2018) and Cushman (2008), Gaboriaud et al. (2022) traced decision processes underlying final judgments using a mouse-tracking tool to measure real-time cognition (see also Gautheron et al. 2023). Tracing underlying dynamics of wrongness and blame judgments is particularly relevant as highlighted by Malle: ‘tracking [of] the time course and accuracy of various ensuing causal and mental inferences, as well as [of] blame itself’ (2021, p. 302). Mouse-tracking can be used in this endeavor, as it allows examining when, in time, the different inputs start and finish exerting their influence during the decision process, before the final judgment is made. Although mouse-tracking has been widely used in social perception (e.g., Freeman & Ambady 2010; Freeman et al. 2011; Smeding et al. 2016) existing evidence is still scarce for moral scenarios (see Gaboriaud et al. 2022; Gautheron et al. 2023; and Koop 2013, for exceptions).

Completing previous studies, the present research focuses on blame and wrongness judgments, which have not been investigated in terms of judgment dynamics and with the direct manipulation of distinguished causality and outcome inputs. This allows directly comparing two of the central types of judgment from Cushman’s and Malle’s views, specifically those involving the highest degree of processing complexity, which is well adapted to the use of mouse-tracking. Further contributing to the moral psychology literature, we used a large sample of moral scenarios, as past research has often relied on a restricted range of scenarios (e.g., Cushman 2008; Kneer & Machery 2019), which threatens broader conclusions to moral psychology in general (Hofmann & Grigoryan 2023). Related to the variability of moral stimuli, previous research did not use mixed models for data analysis, which restricts the findings’ generalizability (to other participants and other moral stimuli). This is an important limitation as the variability between people and between scenarios in the moral domain can account for large portions of variance (e.g., Gaboriaud et al. 2022), hence supporting the use of mixed models. We will use mixed models for all analyses, including participants and stimuli as two random factors.

Regarding confirmatory hypotheses on final judgments, as preregistered, we expected a main effect of intent, outcome, and causality, as well as the intent-by-causality interaction, especially for blame judgments. An intent-by-type of judgment interaction effect was hypothesized, with intent having a stronger influence on judgments of wrongness compared to blame (following the proposed model). Interaction effects between type of judgment and outcome/causality were also expected, with a stronger influence of outcome and causality for judgments of blame compared to judgments of wrongness as illustrated in the model (Figure 1).

Additional exploratory hypotheses were preregistered for judgment dynamics (mouse-tracking data), to replicate, but also to extend previous findings with this methodology (Gaboriaud et al. 2022). When only intent information was available, we merely expected a growing influence of intent from the middle of the trajectory until its end. When intent, causality, and outcome information were all available, we expected an early influence of intent, and a growing influence of outcome and causality during the time course. The effect of intent should, however, remain strong until the end of the process, compared to outcome and causality (following the larger effect size for intent observed on final judgments in previous research; see Cushman 2008; Gaboriaud et al. 2022; Leloup et al. 2018). Although not preregistered, we additionally expected that interaction effects of intent, outcome, and causality factors with type of judgment would be observed concomitantly with the corresponding main effects: from the middle of the trajectory onwards for the intent-by-type of judgment interaction effect when only intent information was available and in a very early stage effect when all information was available; from the middle of the trajectory onwards for outcome and causality-by-type of judgment interaction effects.

## Method

This study was preregistered before data collection. All supplemental materials and data as well as the preregistration form are available on the OSF project page: https://osf.io/675gm/.

### Power Analysis

To determine the required sample size for this study, we ran a power analysis based on the effect sizes and variance partitioning coefficients (VPCs) obtained from a previous experiment with a similar design (see Gaboriaud et al. 2022). We ran this power analysis with the PANGEA online application (version 0.2, Westfall 2016), considering a database of 60 stimuli. Considering three fixed factors (i.e., intent, outcome/causality as one single factor, and type of judgment) and two random factors (i.e., participant and scenario), we needed *n* = 120 participants for the lowest effect size of interest to be detected (i.e., intent-by-causality interaction, *d* = 0.08) with a statistical power of 83%.

For the interaction effects with type of judgment, we could not use information from Cushman (2008) (see further developments provided in the supplemental online materials [SOM]). Therefore, aiming at a power of 80% in a counter-balanced design with 60 stimuli and with VPCs by default, we estimated we could reach a smallest effect size of interest (SESOI) of *d* = 0.28 with 120 participants (calculated with ‘Power Analysis with Crossed Random Effects’ application, see Westfall et al. 2014). Following Lakens’ recommendations (2014), we planned to conduct a sequential analysis. Based on a maximum a priori sample size of 144 participants (i.e., the closest multiple of 48, to guarantee a sufficient sample size for the first interim analysis, and counterbalancing across participants and scenarios), we conducted intermediate analyses at 48 and 96 participants, using a linear spending function (with a general alpha threshold of 0.05). As not all effects of interest were found at these stages, we pursued data collection until reaching *n* = 144.

### Participants

We recruited 145 Psychology undergraduates (131 females, aged from 17 to 40, *M* = 19.97, *SD* = 2.83) who participated in exchange for course credit (one participant was a posteriori removed, see below). Participants provided informed consent to participation. The research was conducted in accordance with the ethical guidelines of the American Psychological Association and was approved by the local ethics committee. All measures, manipulations, and exclusions are disclosed.

### Design

This study followed a variant of a counter-balanced design (i.e., within participant and within stimulus) with three fixed factors (i.e., Intent, Outcome/Causality, and Type of Judgment) and two random factors (i.e., Participant and Scenario). The intent variable was compounded with two modalities: Non-Intentional vs. Intentional, whereas the outcome and causality variables were unified in one single variable [Outcome] defined by a Helmert contrast: Not Caused Harm vs. Caused Harm vs. Neutral Outcome. Finally, the type of judgment (also manipulated within participant and within stimulus) included two modalities: Blame vs. Wrongness (see SOM for more).

### Materials

**Figure 2:**
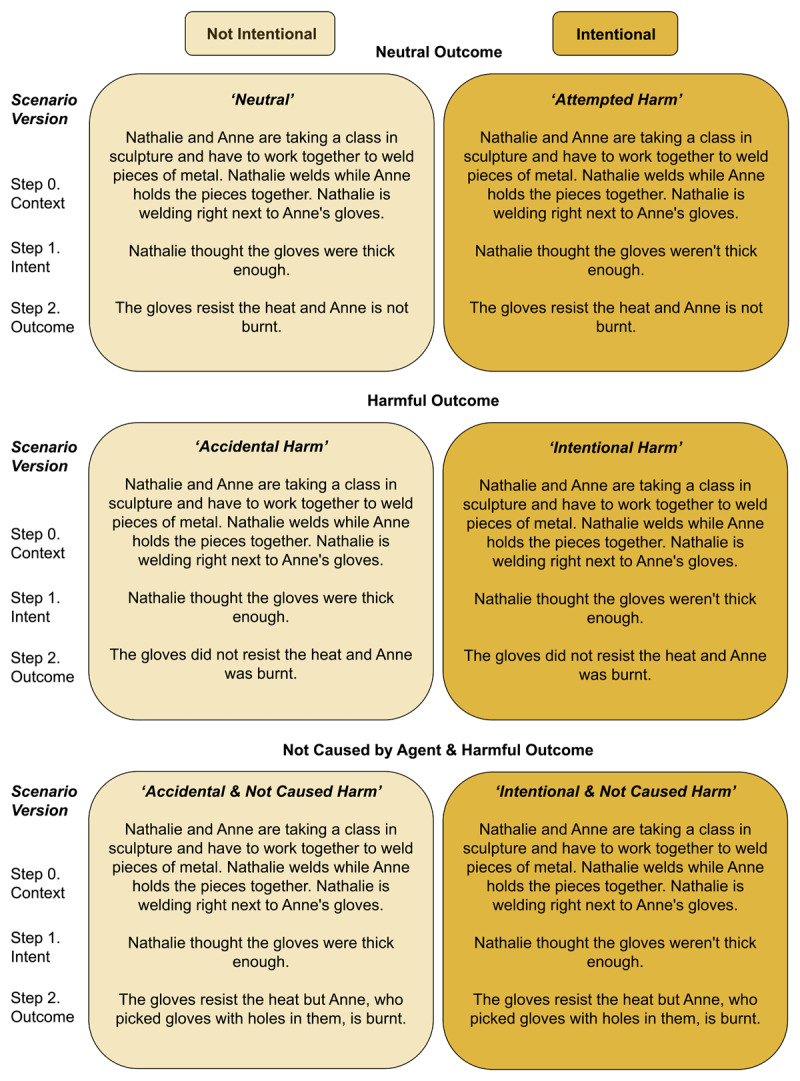
The Six Versions of a Single Scenario, Adapted from Samson and Leloup (2018).

Moral scenarios were adapted from Samson and Leloup (2018, CC-BY 4.0). The 64 French-language scenarios involve each an agent who perpetrates a wrong action with or without intent to do so and a victim who suffers the outcome of the action. Scenarios were further adapted to manipulate the agent’s causal involvement in the outcome (as in Gaboriaud et al. 2022). The gender of the characters was controlled, with only female or male characters in each scenario. See Figure 2 for an example of scenario translated into English in its six possible versions. All judgments were made on a vertical continuous seven-point scale. For judgments of wrongness, it was asked ‘How wrong was the agent’s behavior?’ anchored from 0 (*Not wrong at all*) to 6 (*Very wrong*). For judgments of blame, it was asked ‘How much blame does the agent deserve?’ anchored from 0 (*Not blamable at all*) to 6 (*Very blamable*). These scales were taken from Cushman (2008) but adapted to mouse-tracking. Computer mouse movements over time were recorded as Y-coordinates (on the vertical axis), along with final judgments. These Y-coordinates were rescaled from 0 to 6 to match the judgment response scale. All trajectories were time-normalized from 0% to 100% and trajectories were then decomposed into 20 time bins (Hehman et al. 2015).

### Procedure

**Figure 3:**
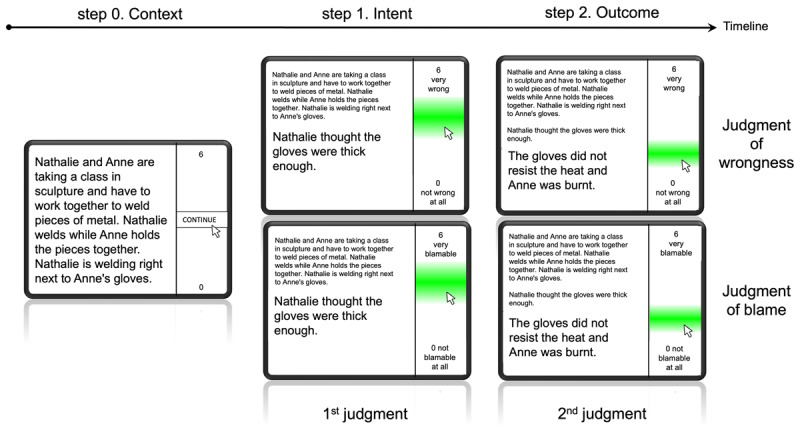
Study’s Procedure Including the Mouse-Tracking Paradigm and Type of Judgment.

Data were collected in France, in a lab environment, and in individual cubicles. After having provided their consent and having filled out demographic information, participants performed four training scenarios. They responded to all scenarios with the computer mouse. We followed the same procedure as Gaboriaud et al. (2022) who embedded moral scenarios within a mouse-tracking paradigm: Participants first went through the context of the moral situation without time limitation (step 0). Then, the information about the agent’s intent was displayed and they gave a first judgment by clicking on the response scale (step 1). After that, the outcome of the action appeared, and participants gave another judgment towards the agent (step 2) (see Figure 3). Participants made judgments of blame or wrongness, depending on the experimental block. The type of block (blame or wrongness) was counterbalanced within participants, for a total of six blocks of ten scenarios per participant. A self-paced break was introduced after each block, which ended with an attention check item (five in total). Participants were then thoroughly debriefed, given their course credit, and thanked.

## Results

### Analytical Strategy

Confirmatory and exploratory analyses were conducted with *R* programming language, *lme4* package (Bates et al. 2015), and some other packages (e.g., *lmerTest, emmeans, car*). Linear mixed models (LMM) were used to generalize results to other participants and moral stimuli (Judd et al. 2012). The intent variable was coded by the following contrast: (–0.5) Non-Intentional vs. (+0.5) Intentional. The outcome and causality variables were unified in one single variable defined by a Helmert contrast. The first one tested the mere presence of a harmful versus neutral outcome; the second contrast tested the agent’s causal involvement in the outcome: Not Caused by Agent (+1/3 or –0.5) vs. Caused by Agent (+1/3 or +0.5) vs. Neutral Outcome (–2/3 or 0). Finally, type of judgment (also manipulated within participant and within stimulus) included two modalities: (–0.5) Wrongness vs. (+0.5) Blame. Final mixed model (after reduction), trial and participant exclusions are fully reported in the SOM for both confirmatory and exploratory analyses. The final sample comprised 144 participants (one participant was excluded because of deviant values in random effects) and the sample of retained trajectories for mouse-tracking analyses was 95% in step 1 and 96% in step 2.

### Confirmatory Results

#### Step 1 Final Judgments

Findings showed a main effect of intent, *b* = 2.94, *SE* = 0.12, *t*(180.69) = 24.90, *p* < .001, 95% CI [2.71,3.18], d = 2.06, with more severe judgments when the agent intentionally perpetrated the wrong action (*M* = 4.44, *SE* = 0.08) than when he or she did not (*M* = 1.49, *SE* = 0.09). Although predicted, we did not find the expected interaction between intent and type of judgment (*b* = –0.07, *SE* = 0.08, *t*(93.34) = –0.89, *p* = .375, [–0.22,0.09], *d* = –0.02). Descriptively, however, means were in the expected direction, with a slightly stronger effect of intent for wrongness (*b* = 2.98) compared to blame judgments (*b* = 2.91). No other main or interaction effects were observed.

#### Step 2 Final Judgments

**Figure 4:**
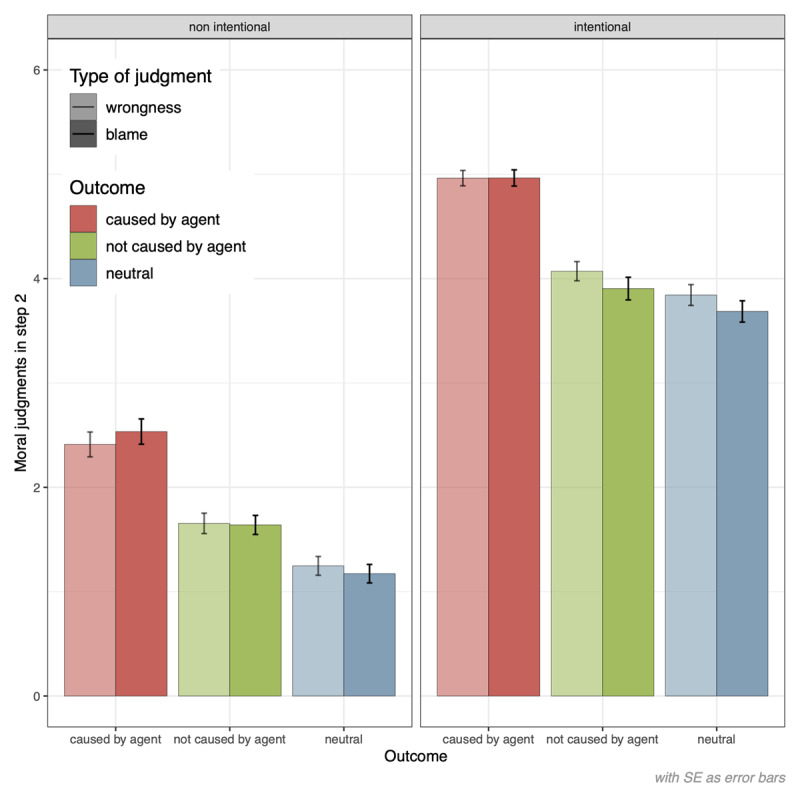
Effects of Intent, Outcome, and Causality Depending on Type of Judgment in Step 2.

Findings showed again the expected main effect of intent, *b* = 2.46, *SE* = 0.10, *t*(176.68) = 23.65, *p* < .001, 95% CI [2.26,2.67], *d* = 1.68 (see Figure 4). We also found a main effect of outcome, *b* = 0.78, *SE* = 0.07, *t*(145.52) = 11.67, *p* < .001, [0.65,0.91], *d* = 0.53, with more severe judgments when there was a harmful (*M* = 3.27, *SE* = 0.06) rather than a neutral outcome (*M* = 2.49, *SE* = 0.07). The main effect of causality was also observed, *b* = 0.90, *SE* = 0.07, *t*(137.37) = 13.26, *p* < .001, [0.77,1.04], *d* = 0.61, with more severe judgments when the agent directly caused the outcome (*M* = 3.72, *SE* = 0.07) compared to when the outcome was caused by another source (*M* = 2.82, *SE* = 0.07) (see Figure 4). The main effect of type of judgment did not reach the retained level of significance, *b* = –0.05, *SE* = 0.03, *t*(7597.60) = –1.89, *p* = .059, [–0.10,0.002], *d* = –0.03, although descriptively, means went again in the direction of slightly more severe wrongness (*M* = 3.03, *SE* = 0.06) than blame (*M* = 2.98, *SE* = 0.06) judgments.

Regarding two-way interactions, an unexpected interaction effect between intent and outcome was observed (*b* = –0.14, *SE* = 0.05, *t*(7439.93) = –2.60, *p* = .009, [–0.24, –0.03], *d* = –0.05), but not between intent and causality at set level of significance (*b* = 0.15, *SE* = 0.08, *t*(88.29) = 1.78, *p* = .079, [–0.02,0.32], *d* = 0.05). These interaction effects parameters went in the opposite direction, indicating that the effect of outcome was stronger in non-intentional (*b* = 0.85) compared to intentional scenarios (*b* = 0.71) whereas the effect of causality was descriptively stronger in intentional cases (*b* = 0.98) compared to non-intentional ones (*b* = 0.85).

The intent-by-type of judgment effect was not significant, *b* = –0.12, *SE* = 0.08, *t*(97.91) = –1.51, *p* = .135, [–0.27,0.04], d = –0.04. However, as hypothesized, the causality-by-type of judgment interaction effect was observed, *b* = 0.15, *SE* = 0.07, *t*(173.97) = 2.05, *p* = .042, [0.006,0.30], *d* = 0.05, such that the causality effect was stronger (+ 0.16) in the case of blame judgments (*b* = 0.98) compared to wrongness judgments (*b* = 0.82). The outcome-by-type of judgment interaction effect was not found (*b* = 0.10, *SE* = 0.06, *t*(163.76) = 1.63, *p* = .105, [–0.02, 0.23], *d* = 0.03). Descriptively, estimated parameters indicated that the effect of outcome was slightly stronger (+ 0.10) for blame (*b* = 0.83) compared to wrongness (*b* = 0.73) judgments. Three-way interactions between intent, outcome/causality, and type of judgment were non-significant (|*t_s_*| ≤ 0.50, *p_s_* ≥ .616).

### Exploratory Results

#### Step 1 Decision Dynamics

In the first part of this experiment, when participants only knew about the general context and the agent’s intent, we detected that the effect of intent became significant only at the end of the time course (around 90%), *b* = 0.13, *SE* = 0.03, *t*(139.20) = 4.10, *p* < .001, 95% CI [0.07,0.20], *d* = 0.18. It became quickly stronger until reaching its maximal value at 100% of the trajectory with *b* = 2.35, *SE* = 0.10, *t*(179.76) = 23.35, *p* < .001, [2.15,2.55], *d* = 1.94. No other stable main or interaction effects were detected in this step.

#### Step 2 Decision Dynamics

**Figure 5:**
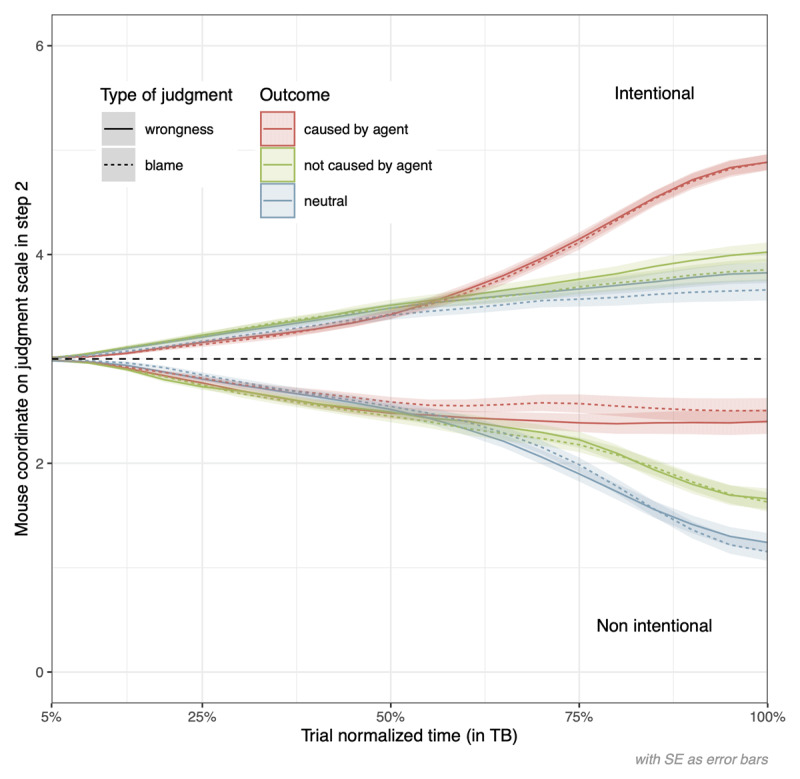
Effects of Intent, Outcome, and Causality Depending on Type of Judgment during Trial Time Course. *Note*. Y-coordinates were rescaled from 0 to 6 (to facilitate comparison with final judgments). Positive (vs. negative) values signaled an attraction toward the more (vs. less) severe judgments.

As participants had already processed intent in step 1, its effect was observed from an early stage onwards (as soon as 0% to 5% of the time course), *b* = 0.02, *SE* = 0.004, *t*(97.28) = 5.86, *p* < .001, 95% CI [0.02, 0.03], *d* = 0.20. It became stronger and reached its maximal value at the end of the trial, *b* = 2.42, *SE* = 0.10, *t*(175.90) = 23.33, *p* < .001, [2.22, 2.63], *d* = 1.67 (see Figure 5). We observed a main effect of causality, which appeared as stable later on, at 60% of the decision process, where it had a weak effect, *b* = 0.09, *SE* = 0.03, *t*(78.95) = 2.72, *p* = .008, [0.02, 0.16], *d* = 0.09). It then became stronger until reaching its maximal value at 100% of trajectory completion, *b* = 0.88, *SE* = 0.07, *t*(136.12) = 12.73, *p* < .001, [0.74, 1.01], *d* = 0.60 (see Figure 5). The expected significant effect of outcome was also observed and appeared as strong and stable from 60% onwards (*b* = 0.08, *SE* = 0.03, *t*(61.87) = 3.08, *p* = .003, [0.03, 0.13], *d* = 0.08). It was already temporarily significant earlier on, from 10% to 35% of the time course (–0.03 ≤ *b_s_* ≤ –0.02, *p_s_* ≤ .035). The outcome effect reached its strongest influence at the end of the process (100%), *b* = 0.76, *SE* = 0.07, *t*(144.30) = 11.30, *p* < .001, [0.63, 0.89], *d* = 0.52 (see Figure 5). Unlike the analysis on final judgments, a main effect of type of judgment was observed, which appeared at the end of the process, around 95%, *b* = –0.06, *SE* = 0.03, *t*(7244.76) = –2.259, *p* = .024, [–0.11, –0.008], *d* = –0.04, with wrongness judgments being more severe compared to blame judgments by the end of the trajectory.

Regarding two-way interaction effects, the intent-by-causality and intent-by-outcome interaction effects were found as significant and stable around 75% of the time course, respectively, *b* = 0.19, *SE* = 0.08, *t*(60.58) = 2.34, *p* = .023, 95% CI [0.03, 0.35], *d* = 0.08 and *b* = –0.09, *SE* = 0.05, *t*(7229.42) = –2.00, *p* = .045, [–0.18, –0.002], *d* = –0.04. They both took their maximal influence at the end of the process (at 80% for the intent-by-causality interaction effect, with *b* = 0.21, and at 95% for the intent-by-outcome interaction effect, with *b* = –0.18). Precisely, and consistent with final judgments, the causality effect became stronger when moving from non-intentionally to intentionally perpetrated scenarios, whereas the outcome effect became weaker during the same move. In addition to this later effect, these interactions also had non-negligible influence earlier in the judgment process, as they were significant as soon as 10% and 15% of the time course until around 55% and 35% respectively. For the intent-by-causality interaction effect, it started at 10%, with *b* = –0.04, *SE* = 0.01, *t*(87.99) = –3.01, *p* = .003, [–0.06, –0.01], *d* = –0.10 and grew until 55% with *b* = –0.12, *SE* = 0.05, *t*(59.39) = –2.26, *p* = .027, [–0.22, –0.01], *d* = –0.06. For the intent-by-outcome interaction effect, it started at 15%, with *b* = 0.04, *SE* = 0.01, *t*(7479.84) = 3.24, *p* = .001, [0.02, 0.06], *d* = 0.07 and grew until 35% with *b* = 0.05, *SE* = 0.03, *t*(7531.70) = 1.97, *p* = .049, [0.0003, 0.11], *d* = 0.04.

Concerning the intent-by-type of judgment interaction effect, it was unstable throughout the process. Particularly, this interaction effect was significant at different moments: from 15% to 25% and from 65% to 80% (–0.14 ≤ *b_s_* ≤ –0.05, .021 ≤ *p_s_* ≤ .048). It signals a stronger effect of intent in wrongness judgments compared to blame judgments at different moments of the judgment process. Consistent with final judgments, the causality-by-type of judgment interaction effect was found significant starting at 60%, with *b* = 0.10, *SE* = 0.05, *t*(229.38) = 1.97, *p* = .050, [0.000009, 0.19], *d* = 0.05, and reached its highest influence at the end (100%) with *b* = 0.15, *SE* = 0.07, *t*(166.39) = 2.02, *p* = .045, [0.004, 0.30], *d* = 0.05. This interaction effect signals that causality took more importance in judgments of blame compared to judgments of wrongness from 60% onwards and became increasingly stronger until the end of the trial. No other two-way interaction effects were observed on dynamics, and the three-way interaction effects were not significant either.

When looking in an exploratory vein at the simple effects of type of judgment within the various conditions of intent and outcome/causality on mouse tracking data, we found a persistent influence of the type of judgment specifically for the intentional and neutral or not caused scenarios, at the end of the trial (around 90%). These simple effects go in the expected direction with more severe wrongness judgments compared to blame ones (see Figure 5). In the non-intentional and caused scenarios, however, there was a persistent significant effect of type of judgment from 30% to 80% of the trajectory as can be seen in Figure 5. This simple effect goes in the direction of more severe blame than wrongness judgments, hence inconsistent with what has been found for the other conditions and with the global (albeit non-significant) type of judgment effect direction on final judgments.

## Discussion

In the current research, we aimed to integrate Malle’s (2021) and Cushman’s (2008) approaches to moral judgment into a novel three-input processing model and to provide a first empirical test of it. In this endeavor, we tested the effects of the three inputs considered (i.e., intent, outcome, and causality), and their interaction effects on moral judgments of wrongness and blame. To examine the temporal influence of these factors on judgment dynamics, a recent adaptation of a mouse-tracking paradigm for moral decision-making was used in the current experiment (see Gaboriaud et al. 2022).

As previously found by Gaboriaud et al. (2022) on judgments of punishment, we first replicated the main effects of intent in steps 1 and 2 (i.e., before and after outcome information was displayed, respectively) and the effects of outcome and causality in step 2 on final moral judgments of wrongness and blame. We did not find clear significant differences between wrongness and blame final judgments, whether in step 1 or step 2. This contrasts with what would have been expected from the findings of Leloup et al. (2018). They found that judgments of wrongness were significantly harsher than judgments of punishment (Leloup et al. 2018, study 2). This result is consistent with findings of Study 1 from Cushman (2008), who found no significant difference between both in terms of final judgment severity (with a between-participant design), but only evidenced different processes implied for each. As judgments of blame and punishment should be very similar in terms of cognitive processes they trigger, as opposed to permissibility and wrongness judgments (Cushman 2008) and regarding Leloup et al.’s findings (2018), we expected to find harsher judgments for wrongness compared to blame, which was again not clearly evidenced on final judgments in the current study (although descriptively, means went in the expected direction with slightly harsher ratings for wrongness than for blame judgments).

Interestingly, however, and thanks to the dynamic measure used to trace the decision temporally (i.e., mouse trajectory coordinates across time), findings signal the influence of type of judgment in step 1 during one-third of the whole decision time course (from 30% to 60%), and at the very end of the trial in step 2. These contrasting results for final judgments versus dynamics may indicate that types of judgment exert their influence during the judgment process, but do not have sufficient weight compared to the other factors involved (i.e., intent and outcome/causality) to shift final judgments. Examining the whole decision process in morality (via dynamic methods such as mouse-tracking) therefore provides initial empirical support to Malle’s call (2021) to take processing (complexity) into account and not only final judgments.

Mouse-tracking data also made it possible to examine the temporal intervention of intent, outcome, and causality factors to compare it with previous findings (especially Gaboriaud et al. 2022). Current findings indicate that intent intervened in the decision process very late in step 1 and very early in step 2 (which is consistent with the fact that participants were already aware of this information at the beginning of step 2). Outcome and causality factors intervened earlier in step 2, around the middle of the time course, with outcome having some unstable influence beforehand. In other words, findings suggest a late influence of intent in step 1, an early influence of outcome in step 2, and then a delayed influence of causality. These results therefore provide only partial support for the temporal intervention of these factors found in previous research (see Gaboriaud et al. 2022, who found an early influence of causality compared to the outcome feature in step 2).

However, to provide a more stringent test of this sequence, all input information may be displayed at the same time. Given mouse-tracking constraints—specifically the requirement to favor quick processing (Hehman et al. 2015) and thus to display limited amounts of information—simultaneous display of all informational features is unrealistic. Adapting mouse-tracking paradigms to moral scenarios is already a risky and rather recent advancement, which certainly explains why only very few studies used it for moral scenarios (see Gaboriaud et al. 2022; Gautheron et al. 2023; Koop 2013, for exceptions). One alternative that may be investigated in future research would be to counterbalance informational features order between participants as done by Leloup et al. (2018), but with a measure of underlying dynamics. Such research, however, may not simultaneously focus on differential effects of types of judgment given additional complexity, which was an important aspect of the present study.

Regarding the expected interaction effects, an interaction effect between intent and outcome but not between intent and causality factors was observed in step 2. Based on the literature (Cushman 2008; Gaboriaud et al. 2022; Leloup et al. 2018), the reverse would have been expected, at least for blame judgments. Gaboriaud et al. (2022) indeed found on judgments of punishment—the closest to blame judgments—that intent interacted with causality but not with outcome (consistently with Cushman’s findings in 2008). When looking exploratorily at the intent-by-outcome and intent-by-causality interaction effects within each type of moral judgment separately, we observed here that the two-way interaction between intent and outcome was significant specifically in the condition of blame. It was, however, not true for intent-by-causality interaction (not significant for any type of judgment).

Moreover, regarding the above-mentioned interaction effects, they went in opposite directions. For the intent-by-outcome interaction effect, parameter estimations indicated that the effect of outcome was less important in intentional than in non-intentional cases. This is consistent with the idea that intent takes most of the variance in such situations, hence a more important weight of outcome when no vile intention from the agent. For the intent-by-causality interaction effect, however, the estimated parameters indicated a slightly more important (non-significant) weight of causality in intentional cases compared to accidental cases. This latest direction is consistent with previous findings (see notably Gaboriaud et al. 2022) but could—regarding the intent-by-outcome interaction direction—indicate that the treatment of causality information may be more linked to the inference of mental states process than outcome-related one (in contrast to Cushman’s 2008 proposition).

Furthermore, when we looked at the interaction effects of these factors with type of judgment, we found evidence for causality, whose effect significantly varied in the expected direction depending on the type of judgment at stake (i.e., more impact in blame than in wrongness judgments). This specific result supports the present new processing model of moral judgment where causality is supposed to get more weight in blame (and punishment) judgments than in the other types of judgment. However, the other two-way interaction effects (i.e., intent-by-type of judgment and outcome-by-type of judgment) did not reach statistical significance. Their respective directions were still consistent with expectations as standing in the proposed model, with slightly more impact of outcome in blame than in wrongness judgments and slightly stronger weight of intent in wrongness compared to blame judgments. These interaction effects directions hence go in line with the present model of moral judgments in terms of the differentiated weights for informational features depending on type of judgment.

When looking in post-hoc analyses at the effect of type of judgment within each intent-by-outcome condition on dynamics, we detected differences between blame and wrongness judgments. They became permanently significant over a good proportion of the trajectory, especially in some conditions. In intentional and not caused or neutral outcome scenarios (i.e., when nothing too crucial happened in terms of consequence or causality), we detected a partially permanent influence of type of judgment in direction of more severe judgments of wrongness than blame (consistently with what was found in final judgment analysis). For non-intentional and caused scenarios, findings signal a persistent difference between blame and wrongness judgments in the middle of the trial but that disappeared at the end. Such influences during the judgment process would not have been detected without the use of a process-tracing method such as mouse-tracking. Even if the three-way interaction effects were not significant globally, these post-hoc results are consistent with the currently developed processing model which poses wrongness as firstly influenced by intent information, yet also by causal information but to a lesser extent and blame firstly influenced by both intent and causal information.

The present findings may also highlight some limitations of Cushman’s methodology (2008) using a between-participant design, comparing results between experiments, and using only a few scenarios to test his model. Cushman (2008) argues that an intentionally perpetrated act would be judged as wrong no matter what outcome follows or who is responsible for the act. Based on the present research using a within-participant design, it seems instead that for both types of judgment (i.e., wrongness and blame), intentional as well as causal features play a role (and a preponderant one) all along the decision process. What differs between the two is more the extent to which they play their role: For wrongness judgments, intent plays a somewhat bigger role than outcome or causality features (even if still non-negligible in weight), whereas, for blame judgments, outcome and causality features play a slightly bigger role than in wrongness (even though intent still has a larger impact). Cushman (2008) also advances that intent and outcome are not supposed to interact whereas intent and causality do (for blame and punishment only). The current result about intent and outcome interacting no matter the type of judgment at stake challenges this finding too.

## Limitations and Perspectives

As reported earlier, we noticed no significant main effect of judgment type on final responses, nor the two-way interaction effects of judgment type with intent or outcome contrary to expectations. This, however, may partially support the alternative view, according to which judgments of blame are rather similar to permissibility and wrongness judgments. In the same logic, Kneer and Machery (2019) found notably blame, permissibility, and wrongness judgments to be roughly similar in terms of their sensitivity to moral luck (i.e., the outcome effect here). Current results indeed show little evidence of an influence of the judgment type on final judgments. However, when looking at mouse trajectories, findings evidence a subtle influence of type of judgment under specific conditions, which was consistent in direction with the proposed integrative model of moral judgment. The current observed differences on final judgments as well as on mouse trajectories remain low in size.

As discussed by Malle (2021), as type of judgment was manipulated in the current study in a within-participant design (even if counter-balanced by block), this may have induced some noise for participants in distinguishing both questions. Malle (2021, Appendix 7) suggests that, in a within-participant design and when plural types of moral judgment are displayed, people may have involuntarily collapsed both questions into one, and then not really differentiated their responses. We cannot ensure that this did not happen, even if we took precautions by rendering explicit the difference between the two questions. Future research may investigate differences in effect sizes when a within versus between-participant design is used.

Concurrently, blame and wrongness judgments also differ in terms of the object they target (Malle et al. 2014). Wrongness focuses more on the moral wrongness of the act itself (independently of who perpetrated it), whereas blame targets more the agent who performed the action (Malle et al. 2014). This may have played in our favor for distinguishing the two questions in a within-participant design, but as the judgment object was not controlled in the current study, we cannot know precisely what the impact of such differentiation of target is.

Another source of variability may be due to the multiplicity of moral scenarios used in the present research and the blurriness relative to the intent part for some scenarios. Indeed, Samson and Leloup’s materials (2018) re-used only the belief component from Cushman’s study (2008) for the intent feature (i.e., subjectively knowing the act could have harmful consequences, without the certainty it will happen), leaving for some situations the reader blurred about whether the agent really anticipated the consequence to occur. However, despite variability in content, the current scenarios mainly focused on immoral acts related to the care/harm moral foundation, as defined by Graham et al. (2011). Results and effect sizes may therefore vary if scenarios are sampled from other foundations or types of moral injury (e.g., purity, loyalty transgressions). Moreover, although variability in scenarios and a within-participant design may add some noise and reduce effect sizes, they favor generalizability not solely to other participants, but also to other moral situations, which was not always warranted in past research using only a very limited number of scenarios (e.g., Cushman 2008; Hofmann & Grigoryan 2023; Kneer & Machery 2019). We may still question how much cultural differences may impact such moral considerations, as the participant sample here considered is a WEIRD sample (i.e., Western, Educated, Industrialized, Rich, Democratic, cf. Henrich et al. 2010). Current findings—as much research in moral psychology—might not generalize to other cultures, but it could therefore be an endeavor for future research with multi-site studies on moral judgment processes.

Finally, what could also be held against the current research is the lack of differentiation between two different questions: the question of the judgment verdict as the final decision and the processes at play for the decision to be taken. Sinnott-Armstrong in his chapter of the Atlas of Morality (2018) urges to better distinguish between verdict and deliberation (i.e., processes at play) but also asks for more precise research questions to put an end to too vague answers. However, what he names ‘deliberation’ or ‘processes’ seems to refer mainly to the brain areas involved in a given moral judgment more than to the dynamic interplay of various inputs as we did. We would thus recommend distinguishing—in addition to deliberation versus verdict—different levels of analysis for deliberation. More precisely, to distinguish deliberation as a process at the neural level (e.g., which brain areas are involved in which judgment), or as a process at the cognitive level (e.g., Cushman’s meaning of a process, for instance, the inference of mental states), and as a process at the information level (i.e., which information inputs intervene and when dynamically during the decision process).

## Conclusion

In the present research, we developed a model of moral judgment combining both Cushman’s (2008) and Malle’s (2021) frameworks. It aims at filling existing shortcomings by integrating the temporality and weight of various features involved in the different types of moral judgment, depending on their complexity. To provide a first empirical test of this three-input processing model, we analyzed the influence of type of judgment, in interaction with three key factors investigated in morality (i.e., intent, outcome, and causality). Beyond the conceptual replication of the main effects of these factors on final judgments, we found no strong evidence for a clear differentiation depending on the judgment type (either wrongness or blame), except for causality. Dynamic data (i.e., mouse trajectories over time), however, allowed us—beyond the partial replication of findings from previous research—to detect some unstable evidence of these interaction effects during specific portions of the trials and specifically for some intent-by-outcome conditions. Present findings hereby partially support the proposed three-input processing model of moral judgments, while also suggesting that the distinction between types of judgment (particularly wrongness versus blame) must not be overestimated.

## Data Accessibility Statement

Data and material used in the current study as well as the preregistration form and supplemental online materials (SOM) are available on the OSF project page: https://osf.io/675gm/.
